# Outcome of Adolescents with Acute Lymphoblastic Leukemia Treated by Pediatrics versus Adults Protocols

**DOI:** 10.1155/2014/697675

**Published:** 2014-11-17

**Authors:** Abeer Ibrahim, Amany Ali, Mahmoud M. Mohammed

**Affiliations:** ^1^Department of Medical Oncology and Hematological Malignancy, South Egypt Cancer Institute, Assiut University, El Methaq Street, Assiut, Egypt; ^2^Department of Pediatrics Oncology and Hematological Malignancy, South Egypt Cancer Institute, Assiut University, Egypt

## Abstract

*Objective.* Several studies showed better outcome in adolescents and young adults with acute lymphoblastic leukemia (ALL) treated with pediatrics protocols than similarly aged patients treated with adults protocols, while other studies showed similar outcome of both protocols. We conducted this study to compare the outcome of our pediatrics and adults therapeutic protocols in treatment of adolescents ALL. *Patients and Methods.* We retrospectively reviewed files of 86 consecutive adolescent ALL patients aged 15–18 years who attended to outpatients clinic from January 2003 to January 2010. 32 out of 86 were treated with pediatrics adopted BFM 90 high risk protocol while 54 were treated with adults adopted BFM protocol. We analyzed the effect of different treatment protocols on achieving complete remission (CR), disease-free survival (DFS), and overall survival (OS). *Results.* The 2 patients groups have almost similar characteristics. The CR was significantly higher in pediatrics protocol 96% versus 89% (*P* = 0.001). Despite the fact that the toxicity profiles were higher in pediatrics protocol, they were tolerable. Moreover, the pediatrics protocol resulted in superior outcome in EFS 67% versus 39% (*P* = 0.001), DFS 65% versus 41% (*P* = 0.000), and OS 67% versus 45% (*P* = 0.000). *Conclusion.* Our study's findings recommend using intensified pediatrics inspired protocol to treat adolescents with acute lymphoblastic leukemia.

## 1. Introduction

Acute lymphoblastic leukemia (ALL) remains one of the most challenging adults' hematological malignancies [1]. With respect to therapy, the use of multiagent chemotherapy regimens for the treatment of acute lymphoblastic leukemia (ALL) is considered as a cancer success story in the pediatric setting [2], which have offered patients who once had a dismal prognosis a cure rate that approaches or exceeds 90% [3, 4]. For adults, the same magnitude of success has not been realized using similar strategies, and the cure rate of adults ALL is estimated to be between 20 and 40% [5, 6]. Adults' patients tend to present with higher risk features at diagnosis, predisposing to chemotherapy resistance and disease relapses after initial achievement of complete remission (CR) [7]. On the other hand, within childhood ALL, older children have shown inferior outcomes, and within adults ALL, younger adults have shown superior outcomes. Retrospective studies focusing on patient's age 15 to 21 years showed that “Adolescents and Young Adults” (AYA) treated with adults ALL protocols have poorer outcomes than similarly aged patients treated with pediatric protocols [8–16]. Five-year event-free survival (EFS) for AYA treated with pediatric regimens ranges from 64% to 69% while in adult regimen it ranges from 34% to 49% [17–20].

In our country, adolescents aged between 15 and 18 years of age are referred either to pediatrics or to adults departments according to physician who firstly made the diagnoses either pediatrician or internist. This study was conducted to assess the outcome of different protocols applied by 2 different teams, namely, pediatrics and adults oncologists in the same age group (adolescent).

## 2. Patients and Methods

### 2.1. Study Eligibility

We retrospectively reviewed files of 86 consecutive adolescent ALL patients aged 15–18 years old attended to outpatients clinic of pediatrics and adults medical oncology and hematological malignancy departments, South Egypt Cancer institute and pediatric oncology department in Sohag Cancer center, Egypt, from January 2003 to January 2010. We divided them into 2 groups according to their different treatment protocols. Group 1 (pediatrics protocol group) included patients treated with the adopted regimen from pediatric BFM90 high risk protocol (BFM90 HR). Since all the patients were above the age of 10, they were all considered as high risk patients (Table 1).

Group 2 (adults protocol group) included patients treated with the adopted regimen from adults BFM protocol (BFM) (Table 2). The 2 protocols were adopted from original protocols by replacing the Daunorubicin (which is not available in our country) with another form of anthracycline. Epirubicin was used in pediatrics protocol whereas Doxorubicin was used in adults' protocol. Also in pediatrics protocol they changed the high dose Methotrexate from 5 mg/m^2^ to 3 g/m^2^ because they found our pediatrics patients cannot tolerate the original dose. All patients enrolled in the study had complete morphological and immunophenotypical data. Patients who previously received antileukemic treatment or had uncontrolled or severe cardiovascular, hepatic, or renal disease not resulting from ALL and/or severe psychiatric condition were excluded. Also we excluded patients with ALL-L3 (Burkitt's-type ALL), t(9 : 22), and T-cell lymphoblastic lymphoma.

The study was approved by the institutional review board, in accordance with the ethical standards of the responsible committee on human experimentation and with the Helsinki Declaration of 1975.

### 2.2. Diagnostic Procedure

Morphologic analysis for bone marrow (BM) and peripheral-blood specimens were stained by May-Grünwald-Giemsa. Immunophenotyping was performed by flow cytometry with monoclonal antibodies reactive with B-(CD10, CD19, CD22, sIg, cIg), T-(CD1, CD2, CD3, CD4, CD5, CD7, CD8), and precursor-cell (TdT, HLA-DR, and CD34)-associated antigens. Chromosomal analyses using FISH on BM samples were performed at diagnosis for t(9 : 22) only.

### 2.3. Treatment and Criteria for Response

The treatment regimens are shown in Tables 1 and 2. Patients who achieved CR received consolidation followed by maintenance for 2 years. Hospitalization, management of infections, and transfusion policies were carried out according to the institutional discretion.

CR was defined as the absence of clinical manifestations of ALL accompanied with neutrophil count higher than 1.5 × 10^9^/L, platelet count higher than 150 × 10^9^/L, and hemoglobin levels higher than 100 g/L and morphological examination of bone marrow shows less than 5% of blast cells.

Patients with blast cells in BM greater than 5% at the end of the induction phase were considered induction failures. Overall survival was defined as the time from diagnosis until date of death or censoring patients alive at last follow-up date. Disease-free survival (DFS) was defined as survival without relapse or death from the date of first CR or censoring patients alive in continuous complete remission at last follow-up date. Event-free survival (EFS) was defined as time from diagnosis to the date failure of induction course, the date of relapse, or death or censoring patients alive in continuous complete remission at last follow-up date [9].

## 3. Statistical Analysis

Bivariate tests, Mann-Whitney test, and variance analysis were used to compare quantitative variables when appropriate and the *X* ^2^ test was used to assess differences in proportions. All comparisons were two-tailed.

Actuarial curves for DFS and OS were plotted according to the Kaplan-Meier method [21] and were compared by the log-rank test. The statistically significant variables identified in univariate analysis were included in multivariable analyses.

## 4. Results

### 4.1. Patient Characteristics

We reviewed retrospectively data of 86 patients who received treatment from January 2003 to January 2010; the characteristics of the patients were summarized in Table 3.

The B lineage was accounted for 81% in pediatrics protocol group and 82% in adults protocol group whereas the T lineage was accounted for 19% in the pediatrics protocols group and 18% in the adults one (*P* = 0.091).

The total leucocytes count (TLC) in B lineage ALL was > 50 × 10^9^ cells/L in 56% in pediatrics protocol group, and 59% in adults protocol group (*P* = 0.049). On the other hand, T lineage ALL showed total TLC count of >100 × 10^9^ cells/L in 30% in pediatrics protocol group, and 33% in adults one (*P* = 0.045). No significant difference was remarked between the 2 groups regarding other variables like median age and distribution of sex.

### 4.2. CR Rates

Our results showed higher percentage of patients who achieved CR in the pediatrics protocol group than the adults' protocol group after first induction (96% versus 89%) (Table 3); only one death was reported during induction in pediatrics protocol group whereas 3 patients died during induction in adults protocol group (*P* = 0.009). No other treatment related deaths were reported in the other phases in both regimens.

Sepsis was the main cause of death in the patient treated with pediatrics protocol and in the 2 patients treated with adults one; however, the third patient in adults protocol group died from CNS hemorrhage.

### 4.3. Relapse Rate

In pediatrics protocol group, 10 patients (31%) had relapsed after median followup of 39 months with cumulative incidence of relapse “CIR” (0.401) and standard error “SE” (0.1), 6 patients (60%) had isolated BM relapse, 2 patients (20%) had CNS relapse, and 2 patients (22%) had testicular relapse. Timing of relapse was as follows: 1 patient relapsed during consolidation phases, 1 patient relapsed during the first of year of maintenance, 2 patients relapsed in the second years of maintenance, 3 patients relapsed after one year of finishing maintenance, and 3 patients relapsed after 2 years of maintenance.

During the same period of followup for the adults protocol group, 30 patients had relapsed (55%) with CIR 0.631 and SE 0.06. 24 patients (80%) had isolated BM relapse, 3 patients (10%) had CNS relapse, 3 patients (10%) had CNS and BM relapse, and none of the patients had testicular relapse. Timing of relapse was as follows: 4 patients relapsed during the consolidation phases, 5 patients during maintenance, 4 patients relapsed shortly after maintenance, and 7 patients relapsed after one year of maintenance, 5 patients after 2 years of finishing maintenance, and 4 patients after 3 years of finishing maintenance.

### 4.4. Dose Intensity

In pediatrics protocol group, the L-asparaginase dose was 8 times higher than the one used in adults protocol. The cytarabine dose was 4 times higher than the adults' doses; the Dexamethasone was double the dose used in adults protocol and the vincristine dose is almost the same in the 2 protocols; however the vincristine was included in maintenance phase in the pediatrics protocol but not in the adults one. The Etoposide, Ifosfamide, and high dose of Methotrexate are included in the pediatrics protocol but not in the adults one. The duration of treatment was longer in the pediatrics protocol than it was in the adults one due to the fact that induction and consolidation take about 8 months in pediatrics protocol, while they take 3.5 months in the adults. Also, the duration of admission to hospital was also longer in pediatric protocol and also supportive treatment was more in pediatric regimen.

### 4.5. Toxicity

The study showed higher incidence grade III and IV neutropenia (Table 4) in pediatrics protocol group which resulted in higher episodes of grade III and IV mucositis; also the frequency of grade III and IV thrombocytopenia was more in pediatrics protocol group. Liver impairment due to L-asparaginase, either in the form of elevated bilirubin levels or in elevated liver enzymes, was significantly higher in pediatrics protocol group. The elevated bilirubin levels occurred in 12 patients (37.5%) in pediatrics protocol group, and in 11 (20.3%) patients in adults protocol group (*P* = 0.001). The elevated liver enzymes occurred in 9 patients (28%) in pediatrics protocol group and in 6 patients (11%) in adults protocol group (*P* = 0.003). However, the liver function tests retained normal levels after median 14 days in pediatric protocol group and 9 days in adults protocol group.

Additionally, there was no significant difference in the number of thromboses related to L-asparaginase that occurred in one patient in pediatric protocol 3% and 2 patients in adults protocol 3.7% in adults group (*P* = 0.091).

### 4.6. Survival Outcome

**Figure 1:**
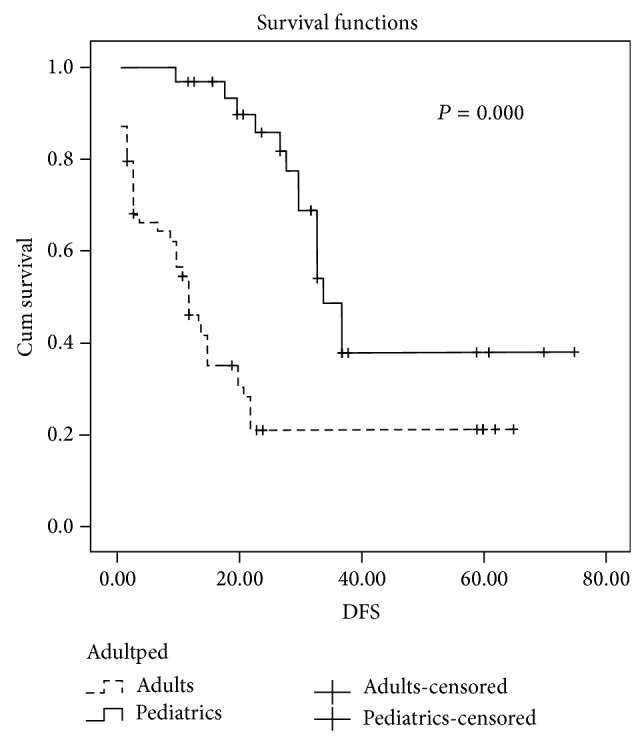
Disease-free survival difference between using pediatrics and adults protocols.

**Figure 2:**
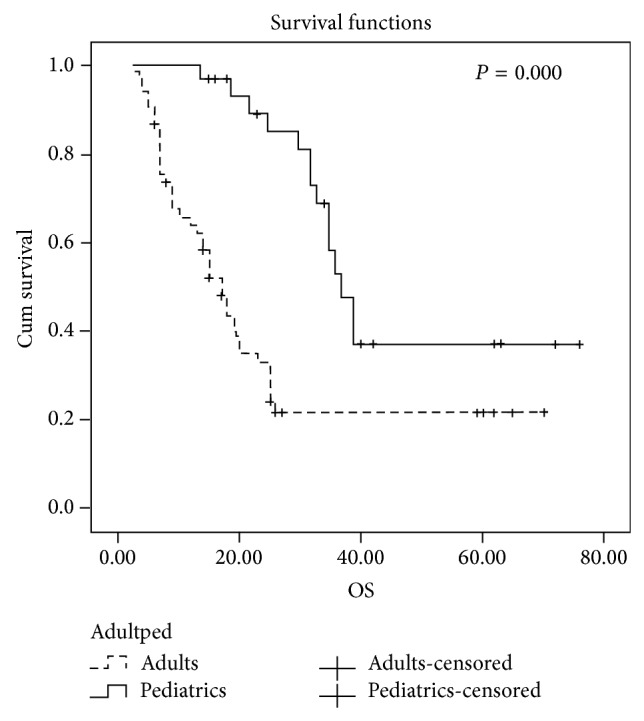
Overall survival difference between using pediatrics and adults' protocols.

After median 39 months of followup, EFS was significantly higher in patients treated in pediatric protocol group 67% (95% CI, 50%–73%) versus 39% (95% CI, 30%–55%) in the adults protocol group *P* = 0.001; the estimated 5-year DFS was 65% (95% CI, 59%–70%) in pediatrics protocol group versus 41% (95% CI, 35%–48%) in adults protocol group *P* = 0.000 (Figure 1). Consequently, OS was higher in pediatric protocol group 67% (95% CI, 60%–72%) than adults protocol group 45% (95% CI, 40%–51%) *P* = 0.000 (Figure 2) (Table 5).

We carried out subanalysis regarding the T lineage groups; the DFS and OS in T-ALL were found to be more than double in pediatrics protocol group compared to that in the adults protocol group 61% versus 25% (*P* = 0.001) and 65% versus 26% (*P* = 0.001), respectively.

## 5. Discussion

Adolescent and young adults AYAs constitute a particular group of patients who find themselves sandwiched between children and adults and who may be referred to either pediatrics or adults oncologists. Several studies comparing the outcome of AYAs on pediatric and adult protocols demonstrated improved survival for AYAs, who were treated by pediatrics protocols; these findings triggered intense interest in the differences with respect to ALL biology and protocol designs in that age group [22–24]. However, because the results are controversial [25], we conducted this study to compare the efficacy and outcome of our institute adopted pediatrics and adults protocols (adult BFM and pediatric BFM90-HR).

The clinical characteristics between 2 groups were quite similar regarding B and T phenotype distribution and the number of patients who had elevated TLC.

Our results showed significant high remission rate 96% and significant difference in EFS (*P* = 0.001) and DFS (*P* = 0.000) in pediatric protocol group. This difference was attributed mainly to high CR rate and lower relapse rate in the pediatrics group. This could be explained based on the differences in induction and consolidation courses, between the 2 protocols since pediatrics protocol has double doses of L-asparaginase and it was repeated in higher doses in all 9 phases of consolidation in pediatrics protocol. Our results are in line with Dana-Farber Consortium study, which showed that children aged 9 to 18 years may have benefited from higher doses of L-asparaginase especially those with T-ALL despite the increased related toxicity [26]. Moreover, the pediatrics protocol is more intensified regimen as it contains higher doses of cytarabine than the adults' protocol in addition to the high doses of Methotrexate which were not included in adults' protocol. The benefit of this strategy was initially proposed by the Berlin-Frankfurt-Munster study group and then it was established by several other studies [27–29]. We noted that the incidence of chemotherapy-related toxicity showed higher significant difference in pediatrics protocol due to the use of more intensified regimen especially in the number of episodes of neutropenia, mucositis, and liver impairment. However, these episodes were reversible and they did not increase the number of deaths. The significant difference which we found in the toxicity profile might contradict the results of similar study conducted by Huguet et al. [20] because they used more intensified regimen in adults than the regimen we used, which resulted in similar toxicity profile when compared to their center pediatrics protocol. Moreover, our results disagree with the results from Finland, which showed no significant difference regarding DFS and OS between their pediatrics and adults protocols; we also found that they used more intensified regimen in adults which was very similar to their pediatrics protocol [25].

## 6. Conclusion

We recommend using intensified pediatrics inspired protocol to treat adolescents with acute lymphoblastic leukemia.

## Figures and Tables

**Table 1:** Adults adopted BFM regimen.

Prephase if (TLC > 2.500 cells/mm^3^ and/or oraganomegaly)			
Vincristine	2 mg	IV	D1
Dexamethasone	10 mg/m^2^	IV	(D1–7)
Phase I induction			
Vincristine	2 mg	IV	(D1, 8, 15, 22)
Doxorubicin	45 mg/m^2^	IV	(D1, 8, 15, 22)
L-asparaginase	5000 u/m^2^	IM	(D15–28)
Dexamethasone	10 mg/m^2^	IV	11 days (if patients received prophase 7 days so to complete 4 more days only)
Methotrexate	15 mg	IT	D1
Phase II induction			
Cyclophosphamide	650 mg/m^2^	IV	(D1, 14, 28)
Cytarabine	75 mg/m^2^	IV	(D3, 4, 5, 6 and 9, 10, 11, 12 and 16, 17, 18, 19 and 23, 24, 25, 26)
Methotrexate	15 mg	IT	Given as 4 weekly (D1, 8, 15, 22)
Cranial prophylaxis			Irradiation (24 Gy)
Phase I consolidation			
Vincristine	2 mg	IV	(D1, 8, 15, 22)
Doxorubicin	45 mg/m^2^	IV	(D1, 8, 15, 22)
Dexamethasone	10 mg/m^2^	IV	For 11 days
Phase II consolidation			
Cyclophosphamide	650 mg/m^2^	IV	(D1, 14, 28)
Cytarabine	75 mg/m^2^	IV	(D3, 4, 5, 6 and 9, 10, 11, 12 and 16, 17, 18, 19 and 23, 24, 25, 26)
Methotrexate	15 mg	IT	4 weekly (D1, 8, 15, 22)
Maintenance will be given for two years			
6-Mercaptopurine	75 mg/m^2^	PO	Daily PO
Methotrexate	20 mg/m^2^	IV	Once weekly
Triple IT cytarabine 40 mg, MTX 15 mg, Dexamethasone 4 mg			Every 2 months till the end of maintenance

D: Day, Gy: Gray, IT: intrathecal, MTX: Methotrexate, PO: per oral, TLC: total leucocytes count.

**Table 2:** Pediatric adopted BFM90 high risk.

Prephase			
Prednisolone	60 mg/m^2^	PO	(D1–7)
Induction			
Prednisolone	60 mg/m^2^	PO	(D1–28)
Vincristine	1.5 mg/m^2^	IV	(D8, 15, 22, 29)
Epirubicin	30 mg/m^2^	IV	(D8, 15, 22, 29)
L-asparaginase	10.000 u/m^2^	IM	(D19, 22, 25, 28, 31, 34, 37, 40)
Triple age adjusted IT		IT	(D8, 15, 22, 29)
High risk I (HRI)			
Dexamethasone	20 mg/m^2^	PO	(D1–5)
Vincristine	1.5 mg/m^2^	IV	(D1–5)
6-Mercaptopurine	25 mg/m^2^	PO	(D1–5)
MTX (6 HR infusion)	3 g/m^2^	IV	(D1)
L-asparaginase	25.000 u/m^2^	IM	(D6)
Triple age adjusted IT		IT	(D1)
High risk II (HRII)			
Dexamethasone	20 mg/m^2^	PO	(D1–5)
Vincristine	1.5 mg/m^2^	IV	(D1)
Ifosfamide	400 mg/m^2^	IV	(D1–5)
MTX (6 HR infusion)	3 g/m^2^	IV	(D1)
Epirubicin	50 mg/m^2^	IV	(D5)
L-asparaginase	25.000 u/m^2^	IM	(D6)
Triple age adjusted IT		IT	(D1)
High risk III (HRIII)			
Dexamethasone	20 mg/m^2^	PO	(D1–5)
Vincristine	5 mg/m^2^	IV	(D1)
Cytarabine	1 g/m^2^/12 h	IV	(D2–5)
Etoposide	150 mg/m^2^	IV	(D2–5)
L-asparaginase	25.000 u/m^2^	IM	(D6)
Triple age adjusted IT		IT	(D1)
Total number of HR are 9 cycles; then if the patient is in CR after the 9th, cranial prophylaxis (18 g) will be given			
		
Maintenance maximum two years with pulses of			
Vincristine	1.5 mg/m^2^	IV	(D1)
Prednisolone	40 mg/m^2^	PO	For 7 days every two months
6-Mercaptopurine	25 mg/m^2^	PO	Daily
MTX	20 mg/m^2^	IM	Weekly
Triple intrathecal (Methotrexate, Ara-C, hydrocortisone)		IT	Every 2 months till the end of maintenance

D: Day, HR: high risk, Gy: gray, MTX: Methotrexate, PO: per oral.

**Table 3:** Patients' characteristics.

Characteristics	Adopted BFM 90 high risk		Adopted BFM for adults		
	Number	%	Number	%	
Sex					
Male	21	66	36	67	NS
Female	11	34	18	33	
Performance status (ECOG)					
0-1	15	47	27	50	0.049
>1	17	53	26		
Median age	16		17		0.931
Phenotype					
B lineage	26	81	44	82	0.999
Early pre-B	4	15	3	7	0.001
Common	4	15	12	27	0.032
Pre-B	18	70	29	66	0.047
T lineage	6	19	10	18	0.919
Total leucocyte count					
Median					
T lineage > 100	2	33	4	40	0.045
B lineage > 50	18	56	26	59	0.049
Serum LDH level					
Normal	7	22	4	6	0.920
Elevated	25	78	50	92	0.051
Induction death	1		3		0.009
CR	27	96	48	89	0.001

CR: complete remission.

**Table 4:** Toxicity profile difference between 2 regimens during induction.

Toxicity	aBFM 90-HR (%)	aBFM standard (%)	*P* value
Neutropenia			
GI-II	60	70	0.049
GIII-IV	40	30	0.049
Thrombocytopenia GIV	40	25	0.003
Mucositis GIII-GIV	30	20	0.005
Thrombotic events	7	6	0.010
Liver impairment			
Elevated bilirubin	37	20	0.001
Elevated enzyme	28	11	0.003

**Table 5:** Survival evaluation according to different protocols.

	aBFM 90-HR (%)	aBFM standard (%)	*P* value
EFS	67	39	0.001
DFS	65	41	0.000
OS	67	45	0.000

CR: complete remission, EFS: event-free survival, DFS: disease-free survival, OS: overall survival.
